# Non-apoptotic function of caspases in a cellular model of hydrogen peroxide-associated colitis

**DOI:** 10.1111/jcmm.12079

**Published:** 2013-06-07

**Authors:** Angela Poehlmann, Kathrin Reissig, Andrea Just, Diana Walluscheck, Roland Hartig, Antje Schinlauer, Wiebke Lessel, Thomas Guenther, Andrew Silver, Pablo Steinberg, Albert Roessner

**Affiliations:** a Department of Pathology, Otto-von-Guericke UniversityMagdeburg, Germany; b Department of Molecular and Clinical Immunology, Otto-von-Guericke UniversityMagdeburg, Germany; c Academic Department of Histopathology, St. Mark's HospitalHarrow, Middlesex, UK; d Colorectal Cancer Genetics, Centre for Digestive Diseases, Blizard Institute of Cell and Molecular Science, Barts and The London School of Medicine and DentistryLondon, UK; e Institute of Food Toxicology and Analytical Chemistry, University of Veterinary Medicine HannoverHannover, Germany

**Keywords:** hydrogen peroxide-associated colitis, DNA-damage checkpoints, non-apoptotic caspase function, JNK-dependent cell cycle arrests, inflammation, neoplastic transformation, ATM degradation, γ-H2AX

## Abstract

Oxidative stress, caused by reactive oxygen species (ROS), is a major contributor to inflammatory bowel disease (IBD)-associated neoplasia. We mimicked ROS exposure of the epithelium in IBD using non-tumour human colonic epithelial cells (HCEC) and hydrogen peroxide (H_2_O_2_). A population of HCEC survived H_2_O_2_-induced oxidative stress *via* JNK-dependent cell cycle arrests. Caspases, p21^WAF1^ and γ-H2AX were identified as JNK-regulated proteins. Up-regulation of caspases was linked to cell survival and not, as expected, to apoptosis. Inhibition using the pan-caspase inhibitor Z-VAD-FMK caused up-regulation of γ-H2AX, a DNA-damage sensor, indicating its negative regulation *via* caspases. Cell cycle analysis revealed an accumulation of HCEC in the G_1_-phase as first response to oxidative stress and increased S-phase population and then apoptosis as second response following caspase inhibition. Thus, caspases execute a non-apoptotic function by promoting cells through G_1_- and S-phase by overriding the G_1_/S- and intra-S checkpoints despite DNA-damage. This led to the accumulation of cells in the G_2_/M-phase and decreased apoptosis. Caspases mediate survival of oxidatively damaged HCEC *via* γ-H2AX suppression, although its direct proteolytic inactivation was excluded. Conversely, we found that oxidative stress led to caspase-dependent proteolytic degradation of the DNA-damage checkpoint protein ATM that is upstream of γ-H2AX. As a consequence, undetected DNA-damage and increased proliferation were found in repeatedly H_2_O_2_-exposed HCEC. Such features have been associated with neoplastic transformation and appear here to be mediated by a non-apoptotic function of caspases. Overexpression of upstream p-JNK in active ulcerative colitis also suggests a potential importance of this pathway *in vivo*.

## Introduction

The most compelling evidence in support of the causal relationship between inflammation and carcinogenesis is provided by ulcerative colitis (UC)-associated colorectal cancer (UC-CRC) and tumours arising as a consequence of Crohn's disease and hepatitis C infection. Indeed, inflammation is regarded as the seventh hallmark of cancer 1. Although it has been suggested that chronic inflammation and colonic injury can directly cause DNA alterations, it remains to be clarified whether inflammation alone without carcinogen exposure can result in tumour initiation 2. In this context, the role of ROS is of major importance 3, particularly as oxidative stress is one of the most important pathogenetic factors in UC-CRC 4, 5.

In a mouse model, cyclical exposure to dextran sulphate sodium (DSS) leads to the development of colitis through induction of epithelial apoptosis, a cellular feature that has also been reported in UC patients 6. However, much less is known about the function of cell cycle arrest in UC and whether arrest links reparative and uncontrolled proliferative response to inflammation. Based on *in vitro* studies, Araki and coworkers suggested that enhanced cell cycle promotion in DSS-induced colitis and UC patients occurs as a reaction following repair from colitis 7. It is well known that cells are provided with DNA-damage checkpoints to control cell cycle progression 8. Overcoming cell cycle control is a fundamental mechanism in the pathogenesis of human cancers. Cells that lack cell cycle control have selective growth advantages. Consequently, genetic changes such as p53 inactivation are important events at the beginning of the UC-carcinoma pathway. It is known that ROS are stress signals for the cell culminating in activation of MAPK's (Mitogen-activated protein kinases), proteins that also play a role in cell cycle checkpoint control 8. Dysregulation of MAPK's and their regulated proteins may, therefore, switch the cellular signalling pathways from cell cycle arrest to enhanced proliferation.

Caspases are cysteinyl-proteases that mediate apoptosis and inflammation *via* proteolytic cleavage of cellular substrates after a specific aspartate residue 9. Novel studies have also shown that caspases have a non-apoptotic function 10–13, including processing of cytokines during inflammation, proliferation of T lymphocytes and terminal differentiation of keratinocytes. In addition, death receptors such as TRAIL-R1/DR4 (TNF-related apoptosis-inducing ligand receptor 1) also execute non-apoptotic functions as they can activate the non-apoptotic NFκB- or JNK pathways *via* the ligand TRAIL 14. Muhlenbeck *et al*. observed caspase-dependent JNK activation, but caspase-independent apoptosis 15. This suggests that the JNK–caspase pathway also unravels a non-apoptotic function of TRAIL.

Herbst *et al*. have shown that the non-tumourigenic human colonic epithelial cell line HCEC 16–21 is suitable to study carcinogen-induced malignant cell transformation 22. Consequently, HCEC is an appropriate cell line to investigate how ROS may act as the link between inflammation and tumourigenesis.

In this study, we aimed at identifying a function of cell cycle arrest in our cellular model of H_2_O_2_-associated colitis. We tried to answer the question of whether arrest may link reparative and uncontrolled proliferative response. We showed that ROS activates the JNK pathway resulting in S- and G_2_/M arrest. Caspases, p21^WAF1^ and γ-H2AX were identified as JNK-regulated proteins. Importantly, caspases executed a non-apoptotic function as they mediated survival of oxidatively damaged HCEC *via* suppression of γ-H2AX. This made the G_1_/S- and intra-S checkpoint ineffective. A population of cells thereby survived. A direct inactivation of γ-H2AX through caspases was excluded. We showed that oxidative stress led to caspase-mediated proteolytic degradation of ATM that is upstream of γ-H2AX. Our findings suggest that delayed arrest in the subsequent cell cycle phases *via* checkpoint override led to survival mediated by a targeting of the caspases by the MAPK/JNK-signalling pathway. We speculate that this survival mechanism during oxidative stress is linked to enhanced proliferation of repeatedly H_2_O_2_-exposed cells in recovery from oxidative stress. The resultant increased proliferation and undetected DNA-damage, both hallmarks of transformation, may serve to initiate tumourigenesis.

## Materials and methods

### Cell culture

For the development of HCEC, a retroviral vector was used to transfer the SV40 large T antigen cDNA into primary HCEC isolated from a non-tumour carrying donor 16. Therefore, HCEC has characteristics consistent with colon, epithelial and non-transformed origin (expression of colon-specific dipeptidyl-peptidase IV, epithelial-specific cytokeratins and no expression of a mutant p53, APC or CEA gene). HCEC cells generated by Nestec Ltd (Nestlé Research Center Lausanne, Switzerland) were obtained from Professor P. Steinberg (Institute of Food Toxicology and Analytical Chemistry, University of Veterinary Medicine Hannover, Germany) and were cultured on collagen-coated plates (1:2000, Becton-Dickinson, Heidelberg, Germany) in basal HCEC cell culture medium (PAN, Biotech GmbH, Aidenbach, Germany) according to Blum *et al*. 16 at 37°C and 5% CO_2_. The basal HCEC medium was supplemented with 2 mM glutamine (PAA, Velizy-Villacoublay, France), 30 μg/ml vitamin C (Roth, Karlsruhe, Germany), 100 nM retinoic acid (Sigma-Aldrich, St. Louis, MO, USA), 1 nM dexamethason (Sigma-Aldrich) and 38 μg/ml bovine pituitary extract (Sigma-Aldrich, Steinheim, Germany) prior to use. In this study, we treated HCEC with pathophysiological H_2_O_2_ concentration (_pat_H_2_O_2_ = 200 μM) as released by macrophages during inflammation 23 to mimic the *in vivo* setting of acute inflammation in colitis. Cells were collected after 24, 48 and 72 hrs after treatment. We generated three altered HCEC cell cultures (HCEC^patH2O2C1-C3^) by three repeated treatments of HCEC with H_2_O_2_ and two recovery phases in between, thus simulating chronic inflammation *via* ROS.

### Inhibition studies

JNK kinase activity was inhibited using the JNK inhibitor SP600125 (Enzo, Lörrach, Germany) at a concentration of 50 μM. The effective inhibition of JNK was ensured through missing phosphorylation of the transcription factor c-jun at serine residues 63 and 73. We inhibited all caspases using the pan-caspase inhibitor Z-VAD-FMK (50 μM, R&D Systems, Minneapolis, MN, USA).

### Cell cycle analysis

One day before treatment, cells were seeded into Petri dishes (90 mm diameter) at a density of 5.0 × 10^5^ cells per dish. For analysis of cell cycle distribution following JNK or caspase inhibition, cells were seeded in 6-well plates at a density of 2.0 × 10^5^ cells per well. Cell cycle analysis was performed as described previously 24. Distribution of cell cycle phases with different DNA contents was determined using a flow cytometer (Calibur, Becton-Dickinson). Cell cycle distribution was analysed, and the percentage of cells in cell debris, G_1_-, S- and G_2_/M-phase of the cell cycle was determined using ModFit software, version LT3.2 (Verity Software House, Topsham, ME, USA).

### Subcellular fractionation

The subcellular fractionation of HCEC and H_2_O_2_-treated HCEC was performed with the Subcellular Protein Fractionation Kit from Thermo Scientific (Rockford, IL, USA) according to the manufacturer's instructions. One to three million cells were separated stepwise, and cytoplasmic, membrane, nuclear soluble, chromatin-bound and cytoskeletal protein extracts were prepared. The extracts were analysed further by immunoblotting.

### Immunoblot analysis

Proteins were prepared as described previously 25. For immunoblot analysis, the following antibodies were used: caspase 3, caspase 8, caspase 9, JNK, p-JNK (Thr183/Tyr185), p-c-jun (Ser63), p-c-jun (Ser73), ATM, cyclin D2, c-fos, p-p38 (Thr180/Tyr182), p-ERK (Thr202/Tyr204), HSP90 (Cell Signaling Technology, Danvers, MA, USA); p21^WAF1^ (Calbiochem, Darmstadt, Germany); p-H2AX (Ser139, γ-H2AX, Millipore, Billerica, MA, USA); β-actin, β-catenin (Sigma-Aldrich); TRAIL-R1/DR4, c-myc (Abcam, Cambridge, UK); H2AX (Upstate, Billerica, MA, USA); CDK6 (Acris, Antibodies, Herford, Germany); GAPDH and H3 (Santa Cruz Biotechnology, Santa Cruz, CA, USA); and Sp1 (Novus Biologicals, Littleton, Colorado, USA). Densitometric analysis of data was performed with the GeneTools Software from Syngene (Cambridge, UK). Fold induction (ratio protein/β-actin) was calculated using the loading control β-actin.

### Proliferation assay

Human colonic epithelial cells and HCEC^patH2O2C1-C3^ were seeded in a 24-well plate at a density of 1 × 10^4^ cells per well. Cells were collected and counted using a particle count and size analyser (COULTER) after 7 days. The cell numbers were determined in quadruplicate.

### Comet assay

To estimate DNA-damage, we performed the CometAssay (Trevigene Gaithersburg, MD, USA) as described previously 24. Evaluation was performed with a fluorescence microscope (Axioplan2, Carl Zeiss Microscopy, Thornwood, NY, USA) equipped with appropriate filter sets. Images were acquired using Isis V 3.4.0 software.

### Phalloidin staining

To characterize filopodia formation of HCEC, cells were cultured in a monolayer on collagen-coated coverslips (Becton-Dickinson) followed by formalin fixation (3.5%, Roth) for 10 min at room temperature. Cells were permeabilized (0.2% TritonX 100, Roth), and non-specific binding was blocked (1% BSA, Roth). F-actin was stained with Alexa Fluor 488 Phallotoxin for 30 min at room temperature (1:200, Invitrogen, Carlsbad, CA, USA). Slides were mounted and nuclei stained with DAPI-mounting medium (Vectashield, Vector Laboratories, Burlingame, CA, USA). Evaluation was performed with the fluorescence microscope (Axioplan2, ZEISS) Isis V 3.4.0 software.

### Human cytokine multiplex assay

To measure cytokine release into the supernatant of HCEC, human multiplex cytokine ELISA (Signosis, Sunnyvale, CA, USA) was used. Therefore, the supernatants of HCEC treated with H_2_O_2_ and DMSO, H_2_O_2_ and SP600125 or Z-VAD-FMK and those of untreated controls were harvested after 72 hrs. Quantitative analyses of interleukins-6, 8 and 13 (Il-6, Il-8, Il-13) and transforming growth factor beta (TGFβ) were performed according to the manufacturer's instructions.

### Immunohistochemistry

The Department of Pathology, Otto-von-Guericke University Magdeburg, Germany provided us with biopsies of intestinal mucosa taken from the regular material entry. The specimens used were taken from the terminal ileum, coecum, colon ascendens, colon transversum, colon descendens, colon sigmoideum and the rectum. So far, one case of UC with high inflammatory activity, two cases of UC in complete remission and three cases of healthy mucosa have been observed. The sections of formalin-fixed and paraffin-embedded colitis specimens or corresponding healthy cases (2.0 μm thick) were mounted on glass slides and dried overnight. The slides were incubated with affinity-purified rabbit antibody against p-JNK (Thr183/Tyr185), (Cell Signaling) diluted 1:50 for 30 min at room temperature, after treatment with 3% H_2_O_2_ for 15 min. The reactions were visualized by DAB detection. The slides were counterstained with haematoxylin and cover slipped after embedding in mounting medium.

### Statistical analysis of data

Student's t-test was used for data analysis. Data are expressed as mean ± SEM, and *P* < 0.05 was considered significant.

## Results

### Oxidative stress led to S- and G_2_/M arrest *via* JNK

**Fig. 1:**
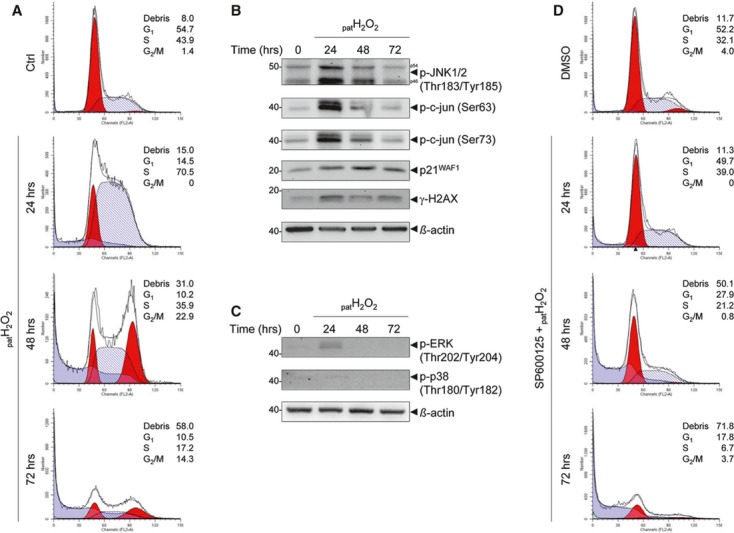
H_2_O_2_ treatment activates DNA-damage checkpoints through the JNK pathway. (**A**) Cell cycle analysis of H_2_O_2_-treated HCEC showed S arrest after 24 hrs, G_2_/M arrest after 48 and 72 hrs and apoptosis after 72 hrs. The data are representative of three independent experiments. (**B**) Activation of the JNK pathway with p-JNK and p-c-jun (Ser63/Ser73) and up-regulation of p21^WAF^^1^. (**C**) H_2_O_2_ did not lead to significant activation of the MAPK's p38 and ERK in HCEC. (**D**) Abrogation of cell cycle arrest following JNK inhibition after 24 to 72 hrs.

Hydrogen peroxide treatment of HCEC caused activation of DNA-damage checkpoints, as S cell cycle arrest was observed after 24 hrs and G_2_/M cell cycle arrest after 48 and 72 hrs (Fig. 1A). At the same time, there was prominent activation of JNK signalling (Fig. 1B), with increased levels of p-JNK and p-c-jun. In addition, up-regulation of p21^WAF1^ and γ-H2AX was detected. Only marginal activation of the other MAPK's p38 and ERK was seen (Fig. 1C). To identify whether the observed arrests are JNK-dependent, the effects of JNK inhibition using SP600125 were analysed (Fig. 1D). After JNK inhibition, H_2_O_2_ nearly completely abolished S arrest after 24 hrs and did not result in cell cycle arrests, but led exclusively to apoptosis after 48 and 72 hrs (Fig. 1D). As the JNK inhibitor was able to rescue S- and G_2_/M arrest, the activation of DNA-damage checkpoints was JNK-dependent. Taking into account that cytokines play a pivotal role in epithelial damage in UC 26, release of several cytokines from H_2_O_2_-stressed HCEC was determined using multiplex assay (Figure S1). We observed H_2_O_2_-dependent induction of Il–13 and TGFβ release. Moreover, Il-6 release and Il-8 release were not significantly affected following H_2_O_2_ treatment.

### Caspases are JNK-regulated proteins and mediate survival of HCEC following oxidative stress

**Fig. 2:**
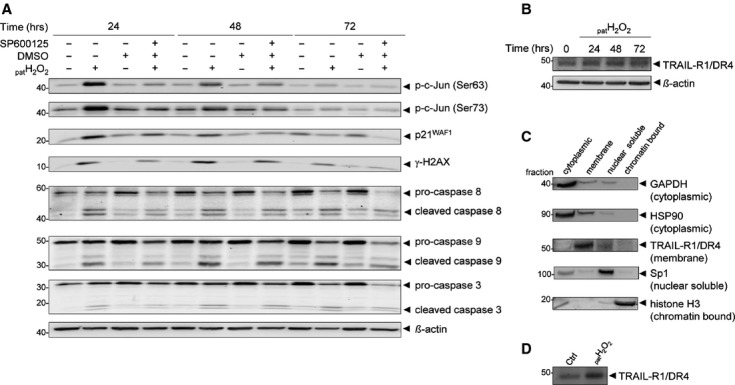
Identification of cellular JNK-regulated proteins in H_2_O_2_-exposed HCEC. (**A**) Immunoblot analysis following JNK inhibition by the JNK inhibitor SP600125 revealed p21^WAF^^1^, γ-H2AX, as well as caspases 3, 8 and 9 as cellular JNK-regulated proteins. The effective inhibition of JNK was ensured through missing phosphorylation of the transcription factor c-jun at serine residues 63 and 73. (**B**) Immunoblot analysis showed an overexpression of the death receptor TRAIL-R1/DR4 after H_2_O_2_. (**C**) Immunoblot analysis of subcellular fractionated cellular proteins of H_2_O_2_-treated HCEC. Each extract was analysed using specific antibodies against proteins from various cellular compartments, including cytoplasmic (GAPDH, HSP90), plasma membrane (TRAIL-R1/DR4), nuclear soluble (Sp1) and chromatin-bound (histone 3). (**D**) Analysis of the membrane extracts of HCEC and H_2_O_2_-exposed HCEC by immunoblotting showed accumulation of the TRAIL-R1/DR4 in H_2_O_2_-exposed cells.

As shown, ROS induced JNK-dependent arrests in HCEC (Fig. 1). In addition, we would like to point out that we observed apoptosis (cell debris: 58% after 72 hrs, Fig. 1A). We detected increased expression of the effector caspase 3 after 24 hrs, and accumulation of active caspase 3 at all three time-points (Figure S2A). However, a broader caspase cleavage was expected to explain the high extent of apoptosis (Fig. 1A). Thus, a crucial involvement of caspases in apoptosis was excluded, and we hypothesized a role of caspases in cellular survival. We found increased expression of caspase 8 and 9 compared with the control 24 hrs after H_2_O_2_ (Fig. 2A), also suggesting induction of their expression. Moreover, we detected an increase in the levels of cleaved caspases 8 and 9 at all time-points in response to H_2_O_2_ (Fig. 2A), which is accompanied by a reduction in the respective uncleaved caspase at 72 hrs as would be expected for cytoplasmic proteins activated *in situ*. To identify JNK-regulated proteins through which JNK influences the cell cycle, we performed immunoblotting analysis following JNK inhibition (Fig. 2A). Proteins were classified as JNK-regulated proteins mediating survival based on down-regulation following JNK inhibition and H_2_O_2_ treatment compared with H_2_O_2_ treatment alone. We found JNK-dependent induction of p21^WAF1^ and γ-H2AX 24 hrs and 48 hrs after H_2_O_2_ treatment respectively. Therefore, we identified p21^WAF1^ and γ-H2AX as JNK-regulated proteins. Moreover, caspase 3, 8 and 9 were down-regulated 24, 48 and 72 hrs, respectively, after JNK inhibition compared with H_2_O_2_. The combined control treatment of H_2_O_2_ and DMSO allocated the observed caspases down-regulation to the JNK inhibitor and not to the solvent (Figure S2B). Thus, we identified caspases 3, 8 and 9 as JNK-regulated proteins that mediate cellular survival. As we also detected decreased cleaved caspase fragments following JNK inhibition, we propose that proteolytic active caspases are involved in survival of HCEC. In addition, cell cycle analysis following JNK inhibition induced increased apoptosis after 72 hrs (cell debris: 72%, Fig. 1D), but no increase in cleaved pro-caspases 3, 8 and 9 (Fig. 2A), further suggesting a non-apoptotic function of caspases.

In addition, we found overexpression of the death receptor TRAIL-R1/DR4 24 and 48 hrs after H_2_O_2_ (Fig. 2B), which could be the cause of a non-apoptotic JNK pathway induction by caspases. Analysis of the membrane fraction after subcellular fractionation of H_2_O_2_-treated HCEC clearly showed the accumulation of TRAIL-R1/DR4 compared with untreated cells (Fig. 2D). Moreover, the release of Il-6 and TGFβ from H_2_O_2_-treated HCEC is JNK-dependent, whereas that of Il-8 and Il-13 seems to be suppressed *via* JNK (Figure S1).

### Non-apoptotic function of caspases through their role in cell cycle progression

**Fig. 3:**
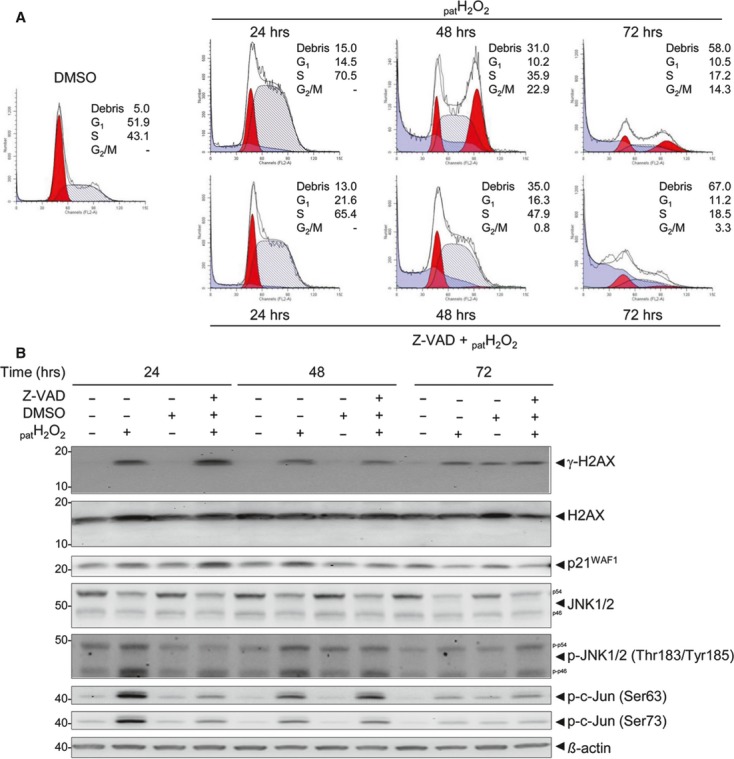
Non-apoptotic function of caspases through their role in cell cycle regulation. (**A**) Cell cycle analysis of H_2_O_2_-treated HCEC following pre-incubation with the pan-caspase inhibitor Z-VAD-FMK showed more cells in the G_1_-phase (24 hrs) and fewer cells in the G_2_/M-phase, but more cells in the G_1_- and S-phase after 48 hrs. After 72 hrs, an apoptotic cell population (cell debris) increased significantly. The data are representative of three independent experiments. (**B**) Immunoblot analysis of H_2_O_2_-exposed HCEC, pre-treated with the pan-caspase inhibitor Z-VAD-FMK, revealed a caspase-mediated negative regulation of γ-H2AX and a positive regulation of the JNK pathway, including p-JNK and p-c-jun (Ser63/Ser73), after 24 hrs.

Cell cycle analysis following caspase inhibition using Z-VAD-FMK showed (*i*) fewer cells in S- and more cells in the G_1_-phase after 24 hrs, (*ii*) fewer cells in G_2_/M- and more cells in the S-, G_1_-phase and debris after 48 hrs and (*iii*) fewer cells in G_2_/M-phase, but more cell debris after 72 hrs (Fig. 3A, G_1_: 14.5–21.6% after 24 hrs, 10.2–16.3% after 48 hrs; cell debris: 58.0–67.0% after 72 hrs). Thus, caspases seem to promote progression of cells through the G_1_- and S-phase by overriding the G_1_/S- and intra-S checkpoint, respectively. Consequently, caspases support cell survival by halting cells in the G_2_/M-phase after 72 hrs. Without proteolytic active caspases, a considerably higher percentage of cells underwent apoptosis (Fig. 3A, cell debris: 67.0% instead of 58.0%). It is apparent, therefore, that apoptosis is caspase-independent and that survival is dependent on proteolytic active caspases, generating cleaved caspase fragments (Fig. 2A).

In summary, caspases promote cell cycle progression and hence survival following oxidative stress by assisting progression of cells through the G_1_- and S-phase.

### Caspases suppress the DNA-damage checkpoint protein γ-H2AX during oxidative stress

To identify caspase-regulated proteins that could mediate progression of cells through G_1_- and S-phase by circumventing of DNA-damage checkpoint control, we performed immunoblot analysis following inhibition of caspase activity (Fig. 3B). As the DNA-damage sensor H2AX phosphorylated at serine 139 is known to play an important role in DNA-damage checkpoint control 27, we investigated its phosphorylation. γ-H2AX was increased significantly 24 hrs after H_2_O_2_- and Z-VAD-FMK treatment paralleled by down-regulation of unmodified H2AX, suggesting negative regulation of H2AX phosphorylation through caspase activity. Thus, caspases seem to mask DNA-damage by negative regulation of H2AX phosphorylation, and this may pave the way to neoplastic transformation as less DNA-repair proteins can be recruited to the site of damage. Caspase-dependent suppression of H2AX phosphorylation may therefore promote progression of cells through the G_1_- and S-phase. Hence, caspase activity seems to circumvent the G_1_/S- and intra-S checkpoint, thereby leading to increased survival despite the presence of DNA-damage. These fundamental factors are likely to contribute to neoplastic transformation of epithelial cells following H_2_O_2_ exposure. Indeed, we detected up-regulation of p21^WAF1^ following inhibition of caspase activity (Fig. 3B). This further supports the idea that sensitive DNA-damage recognition through γ-H2AX led to proper activation of the G_1_/S- and intra-S checkpoint with more cells in the G_1_- and S-phase through p21^WAF1^ up-regulation. As reported by Muhlenbeck *et al*., we also observed caspase-dependent activation of JNK as early response (after 24 hrs) 15, whereas we detected its suppression as second response (after 72 hrs), (Fig. 3B). In addition, the release of Il-13 and TGFβ from H_2_O_2_-treated HCEC was found to be caspase-dependent (Figure S1).

### Caspase-dependent degradation of ATM

**Fig. 4:**
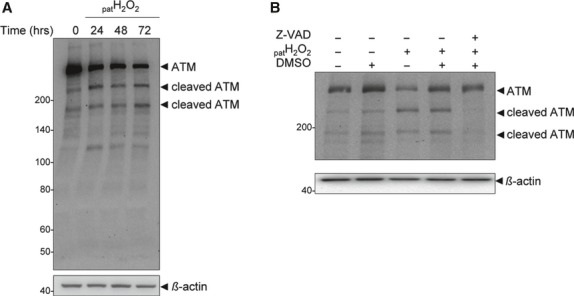
Caspase-dependent degradation of the ATM-kinase. (**A**) Immunoblot analysis showed H_2_O_2_-induced cleavage of ATM after 24, 48 and 72 hrs. (**B**) This cleavage was shown to be reversible after 24 hrs following caspase inhibition (Z-VAD-FMK).

We next proved whether negative regulation of γ-H2AX proceeds *via* its proteolytic degradation. However, cleavage products of H2AX and γ-H2AX could not be observed (Fig. 3B). Therefore, we hypothesized that proteolytic inactivation of proteins occurred upstream of γ-H2AX and that these upstream proteins have a regulatory function by phosphorylating H2AX. Phosphorylation of H2AX at Ser 139 is preferentially catalysed by ATM. Hence, we considered the possibility of caspase-dependent degradation of the upstream molecule ATM. To test this, we first analysed whether oxidative stress may induce proteolytic cleavage of ATM. Immunoblot analysis revealed that ATM is cleaved 24, 48 and 72 hrs following H_2_O_2_ treatment (Fig. 4A). This cleavage was reversible 24 hrs after caspase inhibition (Fig. 4B), confirming caspase-dependent ATM degradation, which resulted in decreased levels of uncleaved ATM (Fig. 4A).

### Masked DNA-damage is linked to γ-H2AX suppression

**Fig. 5:**
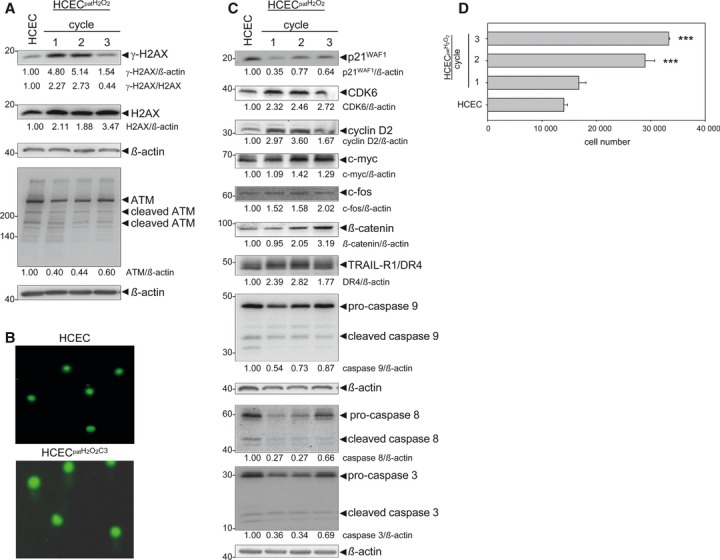
Undetected DNA-damage is linked to γ-H2AX down-regulation. (**A**) Immunoblot analysis of altered HCEC^patH2O2C3^ showed a decreased ratio of γ-H2AX to H2AX, as well as a decreased ATM level in altered HCEC compared with the control. (**B**) Comet assay revealed damaged DNA of HCEC^patH2O2C3^ as enlarged nuclei and comet tails were detected. Untreated HCEC served as control. (**C**) Altered HCEC showed down-regulation of the negative cell cycle regulator p21^WAF^^1^ and up-regulation of the positive cell cycle regulators CDK6 and cyclin D2. Furthermore, an up-regulation of the oncogenic transcription factors c-myc and c-fos as well as of β-catenin and TRAIL-R1/DR4 was detected. Caspases 3, 8 and 9 were found to be down-regulated. (**D**) The repeated exposure of HCEC to H_2_O_2_ led to increased cell proliferation: after 7 days, the number of cells was higher in HCEC^patH2O2C1^ to HCEC^patH2O2C3^ compared with HCEC. Data indicate mean ± SEM and were obtained from four independent experiments. ***P < 0.001.

Chronic UC is characterized by recurrence-remission cycles with periods of mucosal ulceration accompanied by necrosis and regeneration of the colonic mucosa. Accordingly, we simulated the clinical course of IBD with its multiple exposure of colonic epithelial cells to ROS during flares by exposing HCEC to repeated H_2_O_2_ cycles (C1-3) with recovery phases in between exposures (altered HCEC^patH2O2C1-C3^). We then investigated γ-H2AX expression in the newly generated cell cultures, but detected only a marginal protein level in the third cycle, although the non-phosphorylated protein was markedly expressed (Fig. 5A, γ-H2AX : H2AX = 0.44). Hence, the ratio of γ-H2AX to H2AX is lower in HCEC^patH2O2C3^ than in control HCEC. Therefore, there is no significant DNA-damage signalled to the cell's repair mechanisms through γ-H2AX. Importantly, comet assay analysis revealed significantly damaged DNA of HCEC^patH2O2C3^ as indicated by enlarged nuclei, as well as comet tails, which are representative for DNA single- and double-stranded breaks (Fig. 5B). Thus, as it has been suggested, DNA-damage accumulates undetected by the cells because of the negative regulation of γ-H2AX.

### Masked DNA-damage is linked to increased proliferation

**Fig. 6:**
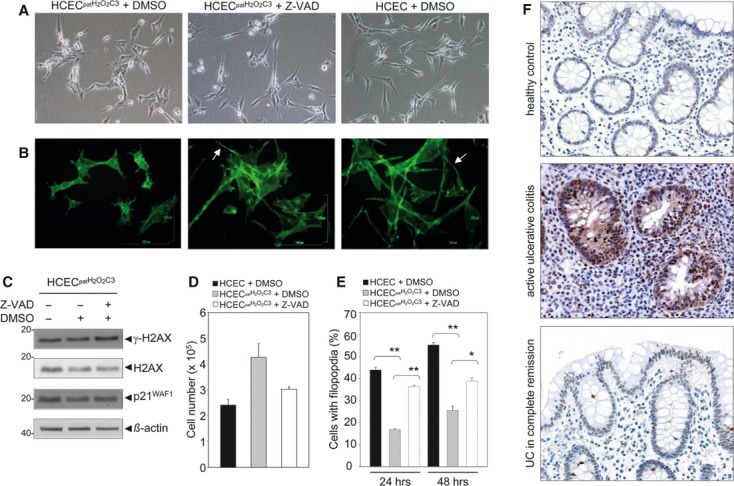
Restoration of the normal HCEC phenotype through caspase inhibition. (**A**) Phase contrast micrographs showed that treatment of HCEC^patH2O2C3^ with the caspase inhibitor Z-VAD-FMK led to the restoration of the morphological HCEC phenotype after 24 hrs. (**B**) FITC-Phalloidin-staining of HCEC^patH2O2C3^, HCEC^patH2O2C3^ treated with Z-VAD-FMK and HCEC. Filopodia are marked. (**C**) HCEC^patH2O2C3^ were treated with caspase inhibitor Z-VAD-FMK, and protein expression of γ-H2AX and p21^WAF^^1^ was analysed after 72 hrs. An increase in both γ-H2AX and p21^WAF^^1^ expression was detected. (**D**) Cell numbers of HCEC, HCEC^patH2O2C3^ and Z-VAD-FMK-treated HCEC^patH2O2C3^ after 72 hrs. The data are representative of three independent experiments. (**E**) Determination of percental filopodia-containing cells of HCEC and HCEC^patH2O2C3^ and of Z-VAD-FMK-treated HCEC^patH2O2C3^ after 24 and 48 hrs. Data indicate mean ± SEM and were obtained from two independent experiments. *P < 0.05, **P < 0.01. (**F**) Immunohistochemical analysis of p-JNK in normal colonic mucosa, active ulcerative colitis (UC) and UC in complete remission.

We next proved whether undetected DNA-damage is linked to increased proliferation. Indeed, we found increased proliferation of HCEC^patH2O2C1-C3^ (Fig. 5D). In this context, we investigated the expression of cell cycle regulators in the newly generated cell cultures. In support of our hypothesis, we detected down-regulation of the negative cell cycle regulator p21^WAF1^ in HCEC^patH2O2C1-C3^, which coincided with up-regulation of the positive cell cycle regulators CDK6 and cyclin D2 compared with the HCEC control (Fig. 5C). Moreover, increased β-catenin levels and increased expression of c-myc and c-fos were found (Fig. 5C), supporting further our hypothesized link to tumourigenesis. We were also able to detect overexpression of the death receptor TRAIL-R1/DR4 and, interestingly, observed down-regulation of caspases in HCEC^patH2O2C1-C3^ (Fig. 5C), which was paralleled by ATM down-regulation (Fig. 5A). However, the decreased ATM levels observed in recovery from oxidative stress (Fig. 5A, altered HCEC^patH2O2C1-C3^) seem to be the result of previous caspase-dependent ATM degradation during oxidative stress. We also detected down-regulation of proteins of the JNK pathway, such as p21^WAF1^. These results are consistent with the observed caspase-dependent suppression of the JNK pathway as second response to oxidative stress (Fig. 3B). Therefore, the JNK pathway, including caspases, represents a non-apoptotic pathway with the potential to link inflammation-associated ROS exposure and tumourigenesis. In this context, as reported by Heckman *et al*. for transformed rat tracheal epithelial cell lines 28, we also observed a more compact shape with decreased filopodia formation as morphological change in HCEC^patH2O2C3^ compared with the normal HCEC phenotype, which is characterized by a flattened shape with filopodial extensions (Fig. 6A and B).

### Restoration of the HCEC phenotype by caspase inhibition

To prove whether caspase activity is linked to a pro-survival function, thus altering the normal HCEC phenotype including its DNA-damage sensitivity, proliferation capacity and morphology, we incubated HCEC^patH2O2C3^ with Z-VAD-FMK. Indeed, we could restore DNA-damage sensitivity of the cells by increasing γ-H2AX levels (Fig. 6C). In addition, we were able to increase p21^WAF1^ expression (Fig. 6C). In line with increased γ-H2AX and p21^WAF1^ expression following caspase inhibition, Z-VAD-FMK-treated HCEC^patH2O2C3^ also showed decreased proliferation 72 hrs following Z-VAD-FMK treatment (Fig. 6D, *P* = 0.051). Furthermore, we could reverse the morphological phenotype of altered HCEC (Fig. 6A and B). As HCEC are characterized by filopodia formation (Fig. 6A), we determined the percentage of cells with filopodia formation of HCEC, as well as that of Z-VAD-FMK-treated HCEC^patH2O2C3^ after 24 and 48 hrs (Fig. 6E). We detected a significant increase in filopodia formation of HCEC^patH2O2C3^ following caspase inhibition (16.9–36.4% after 24 hrs, *P* = 0.001; 25.6–38.9% after 48 hrs, *P* = 0.031). These data support the restoration of the normal HCEC phenotype including its DNA-damage sensitivity, proliferation capacity and morphology by caspase inhibition.

In summary, the results obtained in this study link the non-apoptotic function of caspases to undetected DNA-damage and increased proliferation in this cellular model of hydrogen peroxide-associated colitis. We could also detect overexpression of upstream activated JNK (p-JNK) in active UC by immunohistochemistry as opposed to low activity in both the healthy mucosa and the UC in complete remission (Fig. 6F). These results are in line with our cell culture studies where increased levels of p-JNK have been shown after inflammatory stress (Fig. 1B). This suggests that the results obtained in this cell culture-based study may also reflect events occurring in the *in vivo* situation.

## Discussion

### Oxidative stress activates DNA-damage checkpoints *via* JNK

Constant exposure of cells to DNA-damaging molecules such as ROS during the inflammatory process in IBD is likely to promote tumour development. The well-characterized DNA-damage response mechanisms provide cells with DNA-repair and cell cycle checkpoints. Dysfunction of genes involved in checkpoints and repair are hallmarks of tumourigenesis. A number of proteins are responsible for the control of cell cycle phase progression, including ATM, ATR, Chk1, Chk2 and p53 as the main signalling molecules. Recent evidence also suggests a role for MAPK's in cell cycle checkpoint control, and their dysregulation is potentially related to tumour development 8. MAPK's are known as ROS targets, and the resultant signals are generally coupled to transcriptional activity in the nucleus. However, the function of cell cycle arrest in UC is poorly understood, and it is not known whether arrest links reparative and uncontrolled proliferative response.

We found JNK-dependent cell cycle arrest in HCEC *via* induction of p21^WAF1^ following oxidative stress. In addition, we showed that γ-H2AX is a JNK-regulated protein, implying JNK also a role in DNA-repair. This suggests that JNK may play an important role in linking inflammation to tumourigenesis by linking reparative and uncontrolled proliferative response. Importantly, we also observed an activation of the JNK MAPK's in UC samples. This suggests that besides apoptosis, cell cycle arrest also plays an important role in UC. Recently, the involvement of JNK has been strongly implicated in acute inflammation 29. G_2_/M arrest has also been reported to be JNK-dependent following oxidative stress 30. Based on Araki's study, enhanced cell cycle promotion in DSS-induced colitis in mice and inflammation in UC patients could be a reaction that follows cell cycle arrest 7, but both these observations and the involvement of JNK in the process have to be proven experimentally in some detail.

In addition, colitis ulcerosa is associated with increased cytokine release. This could also be observed in our cellular model of H_2_O_2_-associated colitis for Il-13 and TGFβ, whereas levels of Il-6 and Il-8 remained unchanged. However, the release of Il-6 was found to be JNK-dependent, that of TGFβ was JNK- and caspase-dependent and that of Il-13 was only caspase-dependent. Il-8 and Il-13 were negatively regulated *via* JNK activity. In conclusion, we suggest that Il-13 and TGFβ may play a role in H_2_O_2_-associated colitis. In this context, Kawashima *et al*. recently reported a role of Il-13 in damage of intestinal mucosa 31. TGFβ increased in recovery from oxidative stress, and this fits with the observation that TGFβ depresses mucosal inflammation, promoting tissue repair 32.

Interestingly, H_2_O_2_ treatment caused a transient activation of proteins such as p-JNK p-c-jun, p21^WAF1^ and γ-H2AX with their induction at 24 hrs and their decline at 72 hrs, which is a characteristic sign of recovery. Moreover, JNK levels are decreased following H_2_O_2_ at all time-points. However, the p54 isoforms are diminished more strongly than the p46 isoforms compared with the controls. Caspases 3, 8 and 9 levels also underlay a transient nature with their induction at 24 hrs and their reduction at 72 hrs, which supports their down-regulation in HCEC cycles. However, cleaved caspases levels were still above control level at 72 hrs. This led us to suggest that caspases were activated *in situ*. Moreover, all H_2_O_2_-regulated proteins such as p-c-jun, p21^WAF1^, γ-H2AX and caspases 3, 8 and 9 were JNK-regulated at all time-points.

Interestingly, we found up-regulated TRAIL-R1/DR4 in H_2_O_2_-exposed HCEC and in altered HCEC. Members of the TNF1 receptor are critically involved in inflammatory, immune regulatory and pathophysiological reactions, including the induction of apoptosis 33. However, novel studies show the involvement of this receptor in non-apoptotic pathways such as NFκB- 34 and MAPK-pathway, in particular JNK 15.

### Override of DNA-damage checkpoints links reparative and uncontrolled proliferative response

We found caspases 3, 8 and 9 as JNK-regulated proteins. Our cell cycle analysis of H_2_O_2_-treated HCEC following caspase inhibition showed (*i*) an increased cell population in the G_1_-phase as first response and (*ii*) an increased population of S-phase and then apoptotic HCEC (cell debris) as second response. These results are consistent with the observation that the level of γ-H2AX is increased following caspase inhibition. Thus, we presume that there is an important link between γ-H2AX and the activation of DNA-damage checkpoints that control both induction of cell cycle arrest and apoptosis. This hypothesis is supported by Fragkos *et al*., who reported on the requirement of γ-H2AX for the activation of DNA-damage checkpoints 35. We further suggest that caspases mediate the progression of cells through the G_1_- and S-phase by suppression of H2AX phosphorylation, thereby overriding the G_1_/S- and intra-S checkpoint despite the presence of DNA-damage, and this seems to be the link to survival.

### Caspases-dependent activation of JNK in acute phase and its suppression in chronic phase

We observed caspase-dependent activation of JNK following oxidative stress (24 hrs) and its suppression following recovery from oxidative stress (72 hrs). As caspases expression is induced *via* activated JNK, suppressed JNK activation should cause reduced levels of caspases. Indeed, in recovery from oxidative stress and in the model of chronic exposure, the caspases were reduced in comparison to the control. We found increased proliferation of HCEC^patH2O2C1-C3^ accompanied by down-regulation of p21^WAF1^ and up-regulation of oncogenic transcription factors such as c-myc and c-fos, as well as of β-catenin. The extent of their expression differed in the cycles. However, expression levels of the negative cell cycle regulator p21^WAF1^ were below that of HCEC control, and expression levels of proteins positively triggering proliferation were above that of HCEC control in each cycle, especially those of β-catenin and c-fos in the 3rd cycle. We therefore propose that the interplay of these factors may contribute to increased proliferation of the respective cycle with highest proliferation of the 3rd cycle.

In conclusion, caspases were found to circumvent DNA-damage checkpoints, leading to increased proliferation, decreased DNA-damage sensitivity and changed cell morphology of altered HCEC. However, caspases were down-regulated in HCEC cycles. We therefore presume that not the levels of caspases but rather their activities are crucial.

### Caspases circumvent DNA-damage checkpoints by degradation of the DNA-damage checkpoint protein ATM

Caspases primarily have two functions: (*i*) the processing and activation of pro-inflammatory cytokines and (*ii*) the cleavage of multiple proteins during apoptosis 36. Among the caspases expressed in human cells, caspases 1, 4 and 5 are primarily involved in inflammatory responses by cytokine processing. Caspases 3, 6, 7, 8, 9 and 10 are implicated in apoptosis. Besides these major functions, experimental evidence suggests that caspases might play non-apoptotic roles in processes that are crucial for tumourigenesis such as cell proliferation, migration or invasion 11, 12. Caspases are also involved in the non-proteasomal degradation of inflammatory proteins. Ravi *et al*. reported the repression of NFκB activity in Jurkat T cells following CD95 binding by inducing the proteolytic cleavage of NFκB p65 (RelA) and p50 by caspase-3-related proteases 37. Furthermore, Matthews *et al*. documented a caspase-dependent proteolytic cleavage of STAT3α 38, which may play an important role in modulating STAT3 transcriptional activity. Therefore, it seems that caspases suppress inflammatory signalling by proteolytic degradation of inflammatory proteins such as NFκB and STAT3. This could be a potential link to tumourigenesis following carcinogen exposure.

Studies have highlighted the role of caspases in DNA-damage response. Recent publications suggest that caspase 2 may have a function in response to DNA-damage 39, 40. Indirect evidence that caspase 2 can regulate the cell cycle is based on an association identified between caspase 2 and cyclin D3 41. However, this might provide a link between the cell cycle and cell death.

We could show that oxidative stress led to caspase-mediated degradation of ATM that is upstream of γ-H2AX. The resultant undetected DNA-damage and increased proliferation in altered HCEC may serve to initiate tumourigenesis; both proliferation and DNA-damage are hallmarks of neoplastic change. In addition, we suggest that the increase in γ-H2AX protein levels following caspase inhibition is responsible for increased DNA-damage sensing and G_1_/S- and intra-S-checkpoint activation, and this led to increased G_1_- and S-cell population and increased apoptosis. The proteolytic inactivation of ATM by caspases has recently been reported by Wang *et al*. 42. However, ATM was proteolytically cleaved during cisplatin-induced tubular cell apoptosis.

Overall, loss of functional caspases is not a common event in human cancer, and evasion of apoptosis does not seem to represent a general hallmark of cancer. However, caspases may be involved in tumourigenesis by executing their non-apoptotic functions. To the best of our knowledge, we are the first to demonstrate that caspases might provide a link between cell cycle checkpoint control, cell survival and increased proliferation by proteolytic inactivation of the DNA-damage response protein ATM in HCEC following oxidative stress. Clearly, more work is required to elucidate the functional complexity of caspases in inflammation-associated cancer.

### HCEC are an adequate model to study the link between colonic inflammation and neoplastic transformation

**Fig. 7:**
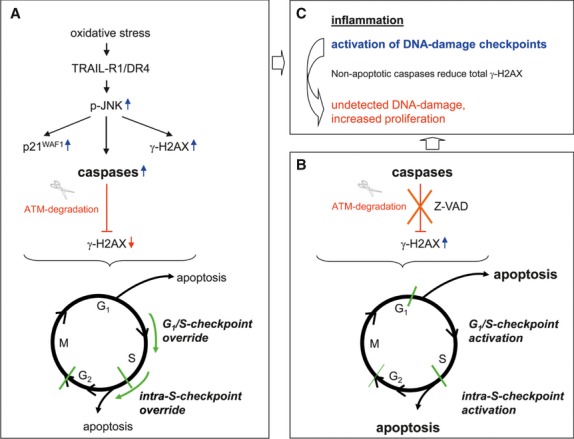
Proposed model of how the non-apoptotic function of caspases allows survival and proliferation of colonic epithelial cells in H_2_O_2_-associated colitis during oxidative stress. (**A**) H_2_O_2_ leads to the induction of the receptor TRAIL-R1/DR4, which, in turn, activates the JNK pathway including p21^WAF^^1^, γ-H2AX and caspases. This results in S and G_2_/M cell cycle arrest. However, caspases are involved in ATM degradation, and this leads to γ-H2AX down-regulation. Caspase activity circumvents G_1_/S- and intra-S checkpoint control by override and progression of cells through the G_1_- and S-phase (survival). (**B**) Inhibition of caspase activity cause γ-H2AX up-regulation and accumulation of cells in G_1_, S, as well as in debris (apoptosis). (**C**) Inflammation-associated ROS cause activation of DNA-damage checkpoints. However, caspase activity causes reduced total γ-H2AX, which leads to undetected accumulation of DNA-damage and increased proliferation of altered HCEC.

We have established a model of how oxidative stress mediates survival of HCEC (Fig. 7):

- Oxidative stress leads to the induction of the TRAIL-Receptor 1/DR4, which, in turn, seems to activate JNK. Consequently, JNK induces the up-regulation of p21^WAF1^, γ-H2AX and caspases, resulting in S and G_2_/M cell cycle arrest. However, caspases are involved in ATM degradation, and this leads to γ-H2AX down-regulation.
- Inhibition of caspase activity revealed an important link between γ-H2AX, G_1_/S- and intra-S-checkpoint activation. Firstly, we observed an increased G_1_-cell population following caspase inhibition, which seems to be the cause of γ-H2AX-mediated G_1_/S-checkpoint activation. Secondly, we detected increased S-cell population and subsequent apoptosis. This suggests γ-H2AX-mediated intra-S-checkpoint activation. Conversely, caspase activity circumvents G_1_/S- and intra-S checkpoint control by override and progression of cells through the G_1_- and S-phase (A). This led to increased proliferation of repeatedly H_2_O_2_-exposed HCEC (C).
- We provide evidence for the importance of DNA-damage checkpoint activation linking reparative and uncontrolled proliferative response through reduced total γ-H2AX. This led to undetected accumulation of DNA-damage and increased proliferation of altered HCEC. Both accumulated DNA-damage and uncontrolled proliferation are hallmarks of neoplastic transformation. In addition, we found the JNK-caspase pathway to be responsible for γ-H2AX regulation. Importantly, caspase-dependent γ-H2AX down-regulation counteracts γ-H2AX up-regulation through p-JNK, which results in a reduction in the total γ-H2AX levels in altered HCEC^patH2O2C3^.

## Supporting information

**Figure S1** H_2_O_2_ causes induction of Il-13 and TGFβ, whereas the Il-6 and Il-8 release remained nearly unchanged. JNK inhibition using SP600125 led to decreased levels of Il-6 and TGFβ, but to increased levels of Il-8 and Il-13. Following inhibition of caspase activity (Z-VAD-FMK), release of IL-13 was abolished, and that of TGFβ was decreased.

**Figure S2** (**A**) H_2_O_2_ results in the up-regulation and cleavage of pro-caspase 3. H_2_O_2_ induced caspase 3 expression after 24 hrs, whereas its expression was reversed after 48 and 72 hrs. (**B**) A solvent effect of DMSO on the decreased caspases expression following JNK inhibition could be excluded. Immunoblot analysis following JNK inhibition by the JNK inhibitor SP600125, compared with the combined DMSO and H_2_O_2_ control, revealed caspases 3, 8 and 9 as JNK-regulated proteins.
