# Supplementary figures and images for: Non-apoptotic function of caspases in a cellular model of hydrogen peroxide-associated colitis
Source: J Cell Mol Med. 2013 Jun 7;17(7):901–13. doi: 10.1111/jcmm.12079 (PMC3822895; doi:10.1111/jcmm.12079)

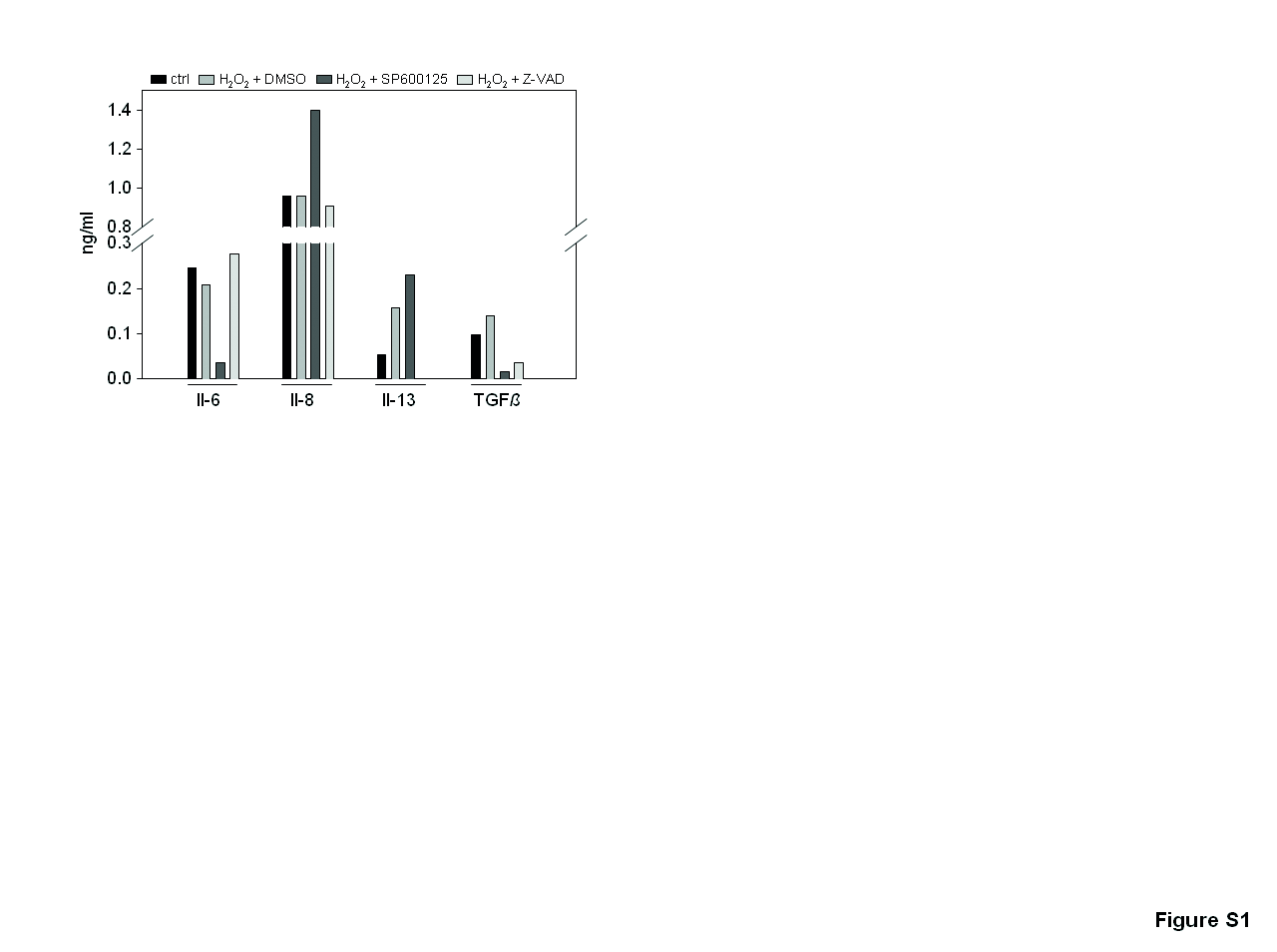

Supplement: Supplementary file 1 [file jcmm0017-0901-SD1.tif]

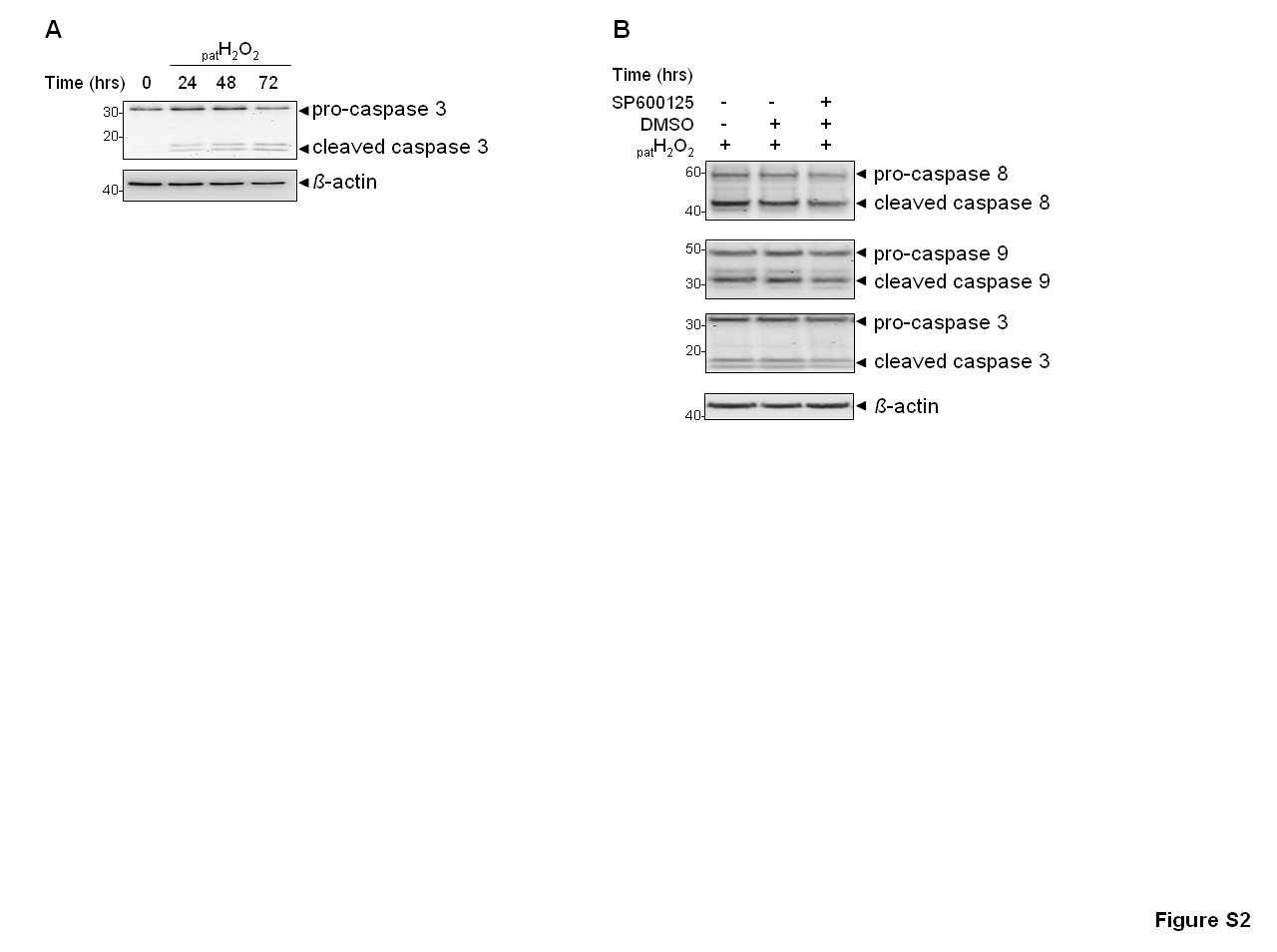

Supplement: Supplementary file 2 [file jcmm0017-0901-SD2.tif]
