# Supplementary material for: Non-apoptotic function of caspases in a cellular model of hydrogen peroxide-associated colitis
Source: J Cell Mol Med. 2013 Jun 7;17(7):901–13. doi: 10.1111/jcmm.12079 (PMC3822895; doi:10.1111/jcmm.12079)
Supplement: Supplementary file 3 [file jcmm0017-0901-SD3.doc]

**Supplementary Figure legends**

**Fig. S1** H_2_O_2_ causes induction of Il-13 and TGF*ß*, while the Il-6 and Il-8 release remained nearly unchanged. JNK-inhibition using SP600125 led to decreased levels of Il-6 and TGF*ß*, but to increased levels of Il-8 and Il-13. Following inhibition of caspase activity (Z-VAD-FMK), release of IL-13 was abolished, and that of TGF*ß* was decreased.

**Fig. S2** (**A**) H_2_O_2_ results in the up-regulation and cleavage of pro-caspase 3. H_2_O_2_ induced caspase 3 expression after 24 hrs, while its expression was reversed after 48 and 72 hrs. (**B**) A solvent effect of DMSO on the decreased caspases expression following JNK inhibition could be excluded. Immunoblot analysis following JNK-inhibition by the JNK inhibitor SP600125, compared to the combined DMSO and H_2_O_2_ control, revealed caspases 3, 8 and 9 as JNK-regulated proteins.
